# Visuomotor Adaptation Brain Changes During a Spaceflight Analog With Elevated Carbon Dioxide (CO_2_): A Pilot Study

**DOI:** 10.3389/fncir.2021.659557

**Published:** 2021-06-07

**Authors:** Ana Paula Salazar, Kathleen E. Hupfeld, Jessica K. Lee, Lauren A. Banker, Grant D. Tays, Nichole E. Beltran, Igor S. Kofman, Yiri E. De Dios, Edwin Mulder, Jacob J. Bloomberg, Ajitkumar P. Mulavara, Rachael D. Seidler

**Affiliations:** ^1^Department of Applied Physiology and Kinesiology, University of Florida, Gainesville, FL, United States; ^2^DLR (Deutsches Zentrum für Luft- und Raumfahrt), Cologne, Germany; ^3^KBR, Houston, TX, United States; ^4^NASA Johnson Space Center, Houston, TX, United States; ^5^Norman Fixel Institute for Neurological Diseases, University of Florida, Gainesville, FL, United States

**Keywords:** sensorimotor adaptation, microgravity, carbon dioxide (CO_2_), head down tilt bed rest, spaceflight

## Abstract

Astronauts on board the International Space Station (ISS) must adapt to several environmental challenges including microgravity, elevated carbon dioxide (CO_2_), and isolation while performing highly controlled movements with complex equipment. Head down tilt bed rest (HDBR) is an analog used to study spaceflight factors including body unloading and headward fluid shifts. We recently reported how HDBR with elevated CO_2_ (HDBR+CO_2_) affects visuomotor adaptation. Here we expand upon this work and examine the effects of HDBR+CO_2_ on brain activity during visuomotor adaptation. Eleven participants (34 ± 8 years) completed six functional MRI (fMRI) sessions pre-, during, and post-HDBR+CO_2_. During fMRI, participants completed a visuomotor adaptation task, divided into baseline, early, late and de-adaptation. Additionally, we compare brain activity between this NASA campaign (30-day HDBR+CO_2_) and a different campaign with a separate set of participants (60-day HDBR with normal atmospheric CO_2_ levels, *n* = 8; 34.25 ± 7.9 years) to characterize the specific effects of CO_2_. Participants were included by convenience. During early adaptation across the HDBR+CO_2_ intervention, participants showed decreasing activation in temporal and subcortical brain regions, followed by post- HDBR+CO_2_ recovery. During late adaptation, participants showed increasing activation in the right fusiform gyrus and right caudate nucleus during HDBR+CO_2_; this activation normalized to baseline levels after bed rest. There were no correlations between brain changes and adaptation performance changes from pre- to post HDBR+CO_2_. Also, there were no statistically significant differences between the HDBR+CO_2_ group and the HDBR controls, suggesting that changes in brain activity were due primarily to bed rest rather than elevated CO_2_. Five HDBR+CO_2_ participants presented with optic disc edema, a sign of Spaceflight Associated Neuro-ocular Syndrome (SANS). An exploratory analysis of HDBR+CO_2_ participants with and without signs of SANS revealed no group differences in brain activity during any phase of the adaptation task. Overall, these findings have implications for spaceflight missions and training, as ISS missions require individuals to adapt to altered sensory inputs over long periods in space. Further, this is the first study to verify the HDBR and elevated CO_2_ effects on the neural correlates of visuomotor adaptation.

## Introduction

During spaceflight, astronauts must adapt to various physiologic challenges including microgravity, elevated carbon dioxide (CO_2_) levels, axial body unloading, and fluid shifts toward the head. These factors can impair sensorimotor function and cognition (De la Torre, 2014). However, it remains unclear whether visuomotor adaptation is impacted by spaceflight. Visuomotor adaptation is a form of sensorimotor learning that requires participants to adapt, or correct for, an external perturbation. Two common examples of visuomotor adaptation include learning to control movements of a computer mouse to accurately move a cursor on a screen under transformed visual feedback and learning to manipulate robotic tools (Rabe et al., 2009). On board the International Space Station (ISS), astronauts must perform highly controlled movements using different types of levers, switches, and various complex scientific equipment. Astronauts thus rely on accurate motor control to appropriately perform these tasks during their day-to-day operations in space.

Typical sensorimotor adaptation tasks involve asking subjects to use a joystick to navigate a computer cursor to a target on a screen; subjects must complete such a task with both accurate and transformed visual feedback. Transformed visual feedback (e.g., rotated cursor feedback or an altered gain of the display) requires subjects to adjust their hand movements to compensate. Such sensorimotor adaptation tasks can be divided into several distinct phases (Smith et al., 2006; Kim et al., 2015). During the baseline phase, individuals receive accurate visual feedback of the cursor position. During the early and late adaptation phases, subjects are presented with a visual perturbation and must adapt their movement accordingly. During the final phase, de-adaptation, the visual perturbation is removed, and individuals are again presented with accurate cursor feedback. In this case, since individuals have adapted to the rotated cursor feedback, they typically show aftereffects and must readapt their movement to readjust to the accurate feedback (Bastian, 2008).

Early adaptation relies on both sensorimotor and cognitive processes (i.e., explicit memory processes); performance improves quickly across several trials during this phase (Anguera et al., 2010; Taylor et al., 2014). In contrast, performance levels are slower to improve during late adaptation (i.e., relying on implicit memory processes; Smith et al., 2006). Previous studies reported that individuals with better spatial working memory also show faster rates of early visuomotor adaptation (Anguera et al., 2010). In addition, past work indicates an overlap in brain activation between the early phase of visuomotor adaptation and spatial working memory task performance; this suggests that the early, but not the late phase of adaptation likely involves spatial cognitive processes (Anguera et al., 2010; Christou et al., 2016).

Head down tilt bed rest (HDBR) is a commonly used spaceflight analog to investigate several effects of the spaceflight environment, including headward fluid shifts and body unloading. Both spaceflight and HDBR are associated with sensory reweighting, changes in cognitive/sensorimotor processes (Bock et al., 2010; Yuan et al., 2016, 2018; Hupfeld et al., 2019), and modifications of brain structure (e.g., Koppelmans et al., 2017; Roberts et al., 2017; Lee et al., 2019b; Hupfeld et al., 2020) and function (Pechenkova et al., 2019) in healthy individuals. Our group (Hupfeld et al., 2019; Lee et al., 2019a; Salazar et al., 2020; McGregor et al., 2021) and others (Zwart et al., 2019; Laurie et al., 2020; Basner et al., 2021) have reported findings from an analog NASA campaign combining HDBR with elevated CO_2_ levels. Compared to HDBR alone, elevated CO_2_ better mimics the ISS environment (~0.4% CO_2_), which has average atmospheric CO_2_ levels approximately 10 times greater than those on Earth. We recently showed that 30 days of HDBR combined with elevated CO_2_ (HDBR+CO_2_) results in reduced brain activity during spatial working memory task performance (Salazar et al., 2020). In another study, we observed adaptive plasticity of the vestibular system during HDBR+CO_2_ followed by recovery after the conclusion of the intervention (Hupfeld et al., 2019). In this work, we found that HDBR+CO_2_ was associated with greater increases in activation of multiple brain regions during vestibular stimulation in comparison with HDBR alone, suggesting interactive or additive effects of bed rest and elevated CO_2_ levels on vestibular processing. However, the effects of HDBR+CO_2_ on the neural correlates of visuomotor adaptation remain unknown.

Our recent work characterizes how the performance of visuomotor adaptation is influenced by HDBR+CO_2_ (Banker et al., 2021). We found that HDBR+CO_2_ alters the way in which individuals engage in sensorimotor processing. Specifically, after 30 days of HDBR+CO_2_, participants showed greater reliance on procedural (i.e., implicit) memory processes during sensorimotor adaptation from pre- to post-intervention (Banker et al., 2021). We also observed declines in early adaptation performance (i.e., greater direction error) from pre- to post-HDBR+CO_2_, as well as slower reaction time during late adaptation that lasted from the last day of HDBR+CO_2_ throughout the two-week recovery period. Moreover, we found no evidence of adaptation savings—faster adaptation that occurs with repeated exposures to the same task—across multiple test sessions. Here we extend this work by using fMRI to characterize brain changes during this visuomotor adaptation task performance across this 30-day HDBR+CO_2_ intervention. In line with our previous fMRI studies using this same cohort of participants (Hupfeld et al., 2019; Salazar et al., 2020; McGregor et al., 2021), we hypothesized that HDBR+CO_2_ would reduce brain activity in regions that are typically involved in sensorimotor adaptation. We anticipated that these changes would begin with the start of HDBR+CO_2_ but recover by 2 weeks post-HDBR+CO_2_.

We addressed three primary aims within this novel pilot study: (1) to investigate the time course of HDBR+CO_2_ effects on brain activation during the four phases of a visuomotor adaptation task; (2) to determine whether any brain changes correlated with changes in visuomotor adaptation performance; (3) to assess the isolated effects of CO_2_ by determining whether any changes in brain activation with 30 days of HDBR+CO_2_ differed from those we observed in a separate study involving 60 days of HDBR with normal atmospheric levels of CO_2_ (referred to here as the HDBR control group).

Five individuals in the HDBR+CO_2_ cohort developed signs of Spaceflight-Associated Neuro-ocular Syndrome (SANS; Laurie et al., 2019). SANS manifests with signs such as optic disc edema and is estimated to affect approximately 16–50 percent of astronauts returning from long-duration missions (Stenger et al., 2017). These individuals showed poorer visuomotor adaptation performance compared to those who did not develop signs of SANS (Banker et al., 2021). Therefore, in the present work, we tested an additional, exploratory aim: (4) to compare subgroup differences between those HDBR+CO_2_ participants who did and did not develop signs of SANS. We will refer to these subgroups as SANS and NoSANS. As mentioned above, the SANS group performed the visuomotor adaptation task slowly (i.e., had longer reaction times) and showed larger, more persistent aftereffects during the de-adaptation phase from pre- to post-HDBR+CO_2_ compared to the NoSANS group. Our previous results suggest that the SANS group may be less aware of the visual-proprioceptive conflict induced by the visuomotor adaptation task and more reliant on implicit adaptation mechanisms (Lee et al., 2019a; Banker et al., 2021). Thus, in the present work we hypothesized that the SANS group would exhibit less brain activity during the early, late, and de-adaptation phases of the task over the course of HDBR+CO_2_ in comparison to NoSANS participants.

## Materials and Methods

### Participants and Testing Timeline

#### HDBR+CO_2_

This longitudinal HDBR+CO_2_ campaign was conducted in 2017 at :envihab, an environmental medicine research facility at the German Aerospace Center (DLR—Deutsches Zentrum für Luft- und Raumfahrt e.V.) in Cologne, Germany. This study included 11 individuals [six males, five females; mean age = 33.91, standard deviation (SD) = 8.03 years]. Participants were tested at six time points: twice before bed rest (“Pre-HDBR+CO_2_ Sessions”—BR-13 and BR-7), twice during the intervention (“HDBR+CO_2_ Sessions”—BR7 and BR29), and twice after the end of bed rest (“Post-HDBR+CO_2_ Sessions”—BR+5 and BR+12). During the 30-day intervention, subjects were restricted to the 6° head-down-tilt position while exposed to ambient 0.5% CO_2_ at all times. They strictly adhered to a controlled diet and 8-hour sleep period (10:30 PM–6:30 AM) and were not allowed to use a regular pillow. Daily activities such as eating, washing, showering, using the toilet, and leisure activities were performed in the 6° head-down tilt position.

Blood samples were collected 3 days prior to bed rest and on the first day after bed rest as part of NASA’s standard measures evaluations; these blood samples allowed for the measurement of the arterial partial pressure of carbon dioxide (PaCO_2_) before and after exposure to the elevated CO_2_ environment.

#### HDBR Control

**Figure 1:**
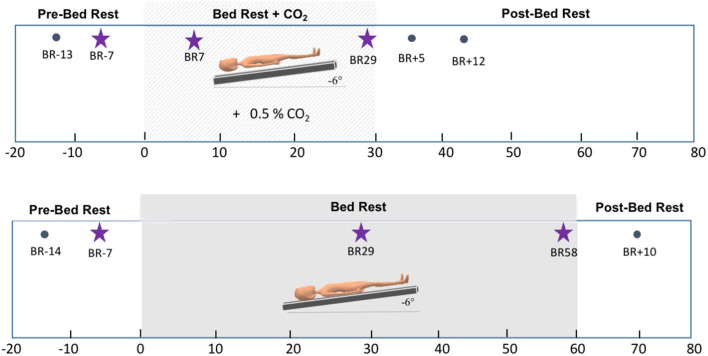
Testing timelines. Top: testing timeline for the HDBR+CO_2_ group. The lighter gray box corresponds to the intervention time, i.e., 30 days of head-down tilt bed rest (HDBR) with 0.5% atmospheric CO_2_. Bottom: testing timeline for the HDBR group. The darker gray box corresponds to the intervention time, i.e., 60 days of HDBR with normal atmospheric CO_2_ levels. Stars indicate the three time points used to create the slope images for between-group comparisons.

We had conducted a separate bed rest campaign in 2019 at the same research facility. This longitudinal study aimed to assess the effects of artificial gravity across 60 days of HDBR as a comprehensive countermeasure against the deleterious effects of spaceflight. In the present work, we examined the participants assigned to the control group of this campaign (*n* = 8; 6 males, 34.25, SD = 7.9 years) to better understand the specific effects of CO_2_ vs. HDBR on brain activity. The HDBR control participants completed identical MRI and behavioral testing protocols compared to the HDBR+CO_2_ group. These participants completed two “Pre-HDBR Sessions” (BR-14 and BR-7) in the 2 weeks prior to starting HDBR. Participants then underwent 60 days of HDBR intervention with normal atmospheric CO_2_ levels. During this time, participants completed two “HDBR Sessions” (BR29 and BR58). Similar to the HDBR+CO_2_ campaign, participants remained supine with a 6° head-down tilt at all times, they were not allowed to use a standard pillow, and they also performed all of their daily activities (e.g., eating, washing, showering, using the toilet, and leisure activities) in the 6° head-down-tilt position. These control subjects stayed at the facility for 14 days after HDBR and completed one recovery data collection session during this time (BR+10). Figure 1 displays details regarding the testing timeline for both groups.

For both of these campaigns, participants provided their written informed consent and received monetary compensation. All procedures were approved by the local ethical commission of the regional medical association in Germany (Ärztekammer Nordrhein) and by the University of Florida and NASA Institutional Review Boards.

### Sensorimotor Adaptation Task

**Figure 2:**
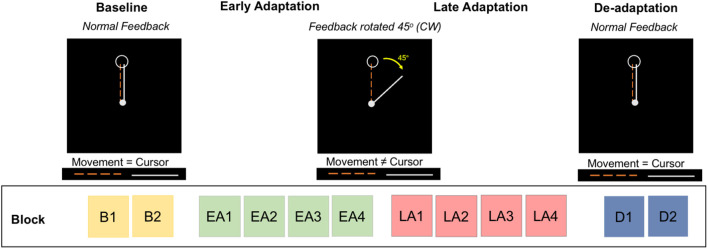
Visuomotor adaptation task. The task includes four phases divided into multiple blocks: baseline (blocks B1, B2), early adaptation (blocks EA1, EA2, EA3, EA4), late adaptation (blocks LA1, LA2, LA3, LA4), and de-adaptation (blocks D1, D2). During baseline and de-adaptation, participants received normal visual feedback from the cursor. In the early and late adaptation phases, participants received a 45° clockwise (CW) rotation of the visual feedback; participants were naïve to this rotation.

At each testing session (Figure 1), participants performed a visuomotor adaptation task in a 3 Tesla Siemens Magnetic Resonance Imaging (MRI) scanner. Figure 2 depicts further details of this task. Participants moved an MRI-compatible joystick with their right thumb and index finger to reach targets presented on a display screen, with real-time feedback of the joystick location presented as a cursor on the screen. At the beginning of each trial, both a home position target and the cursor appeared in the center of the screen (Figure 2). A target (i.e., an open circle) then appeared in one of four positions located to the right, left, above, or below the origin. Participants were instructed to: (1) move the cursor towards the target as quickly as possible, (2) hold the cursor within the target circle until it disappeared, and then (3) release the joystick to allow the cursor to re-center to the initial start position.

The task consisted of four phases: (1) baseline, (2) early adaptation, (3) late adaptation, and (4) de-adaptation. The baseline phase comprised two blocks (B1 and B2). During baseline, participants received real-time veridical visual feedback of the cursor position. During early (blocks EA1, EA2, EA3, EA4) and late (blocks LA1, LA2, LA3, LA4) adaptation, the visual feedback of the cursor location was rotated 45° clockwise (CW) around the central start location; participants did not receive explicit instructions. This visual transformation was introduced to induce an adaptive response; participants gradually adapt to this transformation across trials. During de-adaptation (blocks D1 and D2), the 45° visual perturbation was removed, and participants again received veridical visual feedback of cursor position. This phase of the task allowed us to measure the aftereffects of adaptation.

Similar to our past work (Ruitenberg et al., 2018; Banker et al., 2021), we used direction error as performance outcome metric which was defined as the angle between the line connecting the cursor and the target position (joystick coordinates) at the beginning of the trial and the line from the cursor’s start position to its position (joystick spatial location) at the time of peak velocity. Participants completed a total of 192 trials across the four task phases (e.g., 16 trials per block), at each testing session, however, we analyzed 164 out of 192 trials (i.e., the last 2 or 3 trials of each block were excluded) to be consistent with the number of trials performed by astronauts aboard the ISS in our ongoing longitudinal spaceflight study (Koppelmans et al., 2013; Banker et al., 2021).

### fMRI Acquisition Parameters

Neuroimaging data were acquired on a 3-Tesla Siemens Biograph mMR scanner located at the :envihab facility. Identical fMRI acquisition parameters were used for the HDBR+CO_2_ and HDBR control groups. A gradient echo T2*-weighted echo-planar imaging sequence with the following parameters to acquire fMRI data: TR: 2500 ms, TE: 32 ms, flip angle: 90°, FOV: 192 × 192 mm, matrix: 64 × 64, slice thickness: 3.5 mm, voxel size: 3 × 3 × 3.5 mm^3^, 37 slices. We also acquired a T1-weighted gradient-echo pulse sequence with the following parameters: TR: 1.9 s, TE: 2.4 ms, flip angle: 9°, FOV: 250 × 250 mm, matrix: 512 × 512, slice thickness: 1.0 mm, voxel size: 0.49 × 0.49 × 1.0 mm^3^, 192 slices. During the intervention (“HDBR+CO_2_ Sessions”), the 0.5% CO_2_ level was maintained during scan sessions with a mask and tank system. During all testing sessions, the subject lay on a foam wedge to maintain the 6° HDBR body posture, although the head was supine within the MRI head coil.

Subjects performed the visuomotor adaptation task in a block design with alternating blocks of two conditions, task (40 s) and rest (20 s). The task was repeated for four blocks of adaptation trials labeled as “early adaptation”, and for another four labeled as “late adaptation”.

### fMRI Data Preprocessing and Statistical Analyses

#### Whole Brain Preprocessing

We performed fMRI preprocessing and statistical analyses using Statistical Parametric Mapping 12 (SPM12, version 7219), MATLAB R2018a, version 9.0, and Advanced Normalization Tools (ANTs; Avants et al., 2011). First, we corrected the functional images for slice timing, and realigned and resliced the images to correct for volume-to-volume head motion. Next, we performed an additional quality check using the Artifact Detection Tool (ART^1^). We covaried out volumes with a motion threshold equal to or greater than 2.5 mm and a global brain signal Z threshold equal to or greater than nine. Next, we normalized the whole brain fMRI to Montreal Neurologic Institute 152 (MNI152) standard space using ANTs (Avants et al., 2011), in a multi-step procedure: (1) the T1 images were skull stripped using ImCalc (SPM12); (2) participant-specific T1 templates were created using ANTs’ AntsMultivariateTemplateConstuction.sh function; (3) then, to normalize the functional images to standard space, we (4) created participant-specific mean fMRI templates (again using ANTs’ AntsMultivariateTemplateConstuction.sh function); (5) mean fMRI templates were coregistered to the T1 participant-specific templates using AntsRegistration.sh; (6) the T1 templates were normalized to MNI152 standard space using ANTs’ AntsRegistration.sh function; and (7) the resulting warp parameters were applied to the fMRI using ANTs’ AntsApplyTransforms.sh function. Finally, using SPM12, the normalized fMRI was spatially smoothed with an 8 mm full-width half-maximum three-dimensional Gaussian kernel. This preprocessing procedure is identical to that used in our past HDBR neuroimaging work (Hupfeld et al., 2019; Salazar et al., 2020).

#### Cerebellum Preprocessing

We applied specialized preprocessing to the cerebellum using portions of both the CEREbellum Segmentation (CERES; Romero et al., 2017) pipeline and the Spatially Unbiased Infratentorial Template (SUIT; Diedrichsen, 2006; Diedrichsen et al., 2009) pipeline, again in an identical manner to our past HDBR work (Hupfeld et al., 2019; Salazar et al., 2020). We used these specialized processing algorithms because whole brain warping to a standard MNI template has been found to distort cerebellar structures (Diedrichsen, 2006; Diedrichsen et al., 2009). First, we used the CERES pipeline to segment the cerebellum from each T1-weighted image. We then reset the origin of each individual’s cerebellum segmentation in native space to allow us to coregister each subject’s native space segmentation to the SUIT.nii template. Next, we created binary gray matter, white matter, and full cerebellar masks from the CERES native space output and used the suit_normalize_dartel function to obtain the affine transformation matrix and flowfield needed to normalize these images into SUIT space.

We then coregistered the slice time corrected realigned (but not MNI-normalized) whole brain images to the whole brain T1-weighted images in native space. We re-ran the subject-level statistical analyses described below (Section Subject-Level Statistics) on these *non-normalized* whole brain images. Then, using the Affine transformation matrix and flowfield from normalizing the structural cerebellar segments to SUIT space, as well as each subject’s native space full cerebellar mask, we applied suit_reslice_dartel to the whole brain functional images to reslice all of these images into SUIT space. We then applied a 2 mm full-width half-maximum three-dimensional smoothing Gaussian kernel to the SUIT-normalized cerebellar statistical images.

#### Subject-Level Statistics

We calculated subject-level brain activity during visuomotor adaptation separately for the whole brain and for the cerebellum. We produced four statistical maps for each participant and time point on a voxel-by-voxel basis using the following contrasts: baseline > rest, early adaptation > rest, late adaptation > rest, and de-adaptation > rest. As in our past work (Hupfeld et al., 2019; Salazar et al., 2020), we set the first level masking threshold to-Infinity instead of the default SPM masking threshold of 0.80 and masked out non-brain areas using the SPM intracranial volume mask. This allowed for the inclusion of all voxels in the first-level general linear model (GLM), whereas the SPM default includes in the GLM only those voxels with a mean value ≥ 80% of the global signal. The following nuisance covariates were used in the subject level analyses: the first time derivative of the hemodynamic response function, the SPM-derived head motion parameters (X, Y, and Z translations and roll, pitch, and yaw rotations), as well as the outliers from ART described above (Hupfeld et al., 2019; Salazar et al., 2020).

### fMRI Group-Level Statistical Analyses

#### Main Effect of Adaptation

We used the Sandwich Estimator (SwE) SPM toolbox defaults except for nonparametric wild bootstrap with 999 bootstraps and threshold-free cluster enhancement (TFCE, Smith and Nichols, 2009) to calculate the main effect of each phase of the task for each group. For the HDBR+CO_2_ group, we averaged all subjects (*n* = 11) and sessions BR-7, BR7, and BR29. For HDBR control group we also averaged all subjects (*n* = 8) and sessions BR-7, BR29, and BR58. TFCE does not require an arbitrary cluster-forming threshold and is more sensitive compared to other thresholding methods (Smith and Nichols, 2009).

For this model and for the models described below, we set statistical significance at peak-level familywise error (FWE) <0.1, which is a lenient threshold, though it accounts for multiple comparisons. We accepted clusters that were larger than 10 voxels for the whole brain analyses and larger than five voxels for the cerebellum analyses. We included mean-centered age and sex as covariates of no interest.

#### Time Course of Neural Visuomotor Adaptation Response to HDBR+CO_2_

Identical to our past work (Yuan et al., 2018; Hupfeld et al., 2019; Salazar et al., 2020), to test for brain regions that showed a pattern of cumulative change followed by post-HDBR+CO_2_ recovery we tested several *a priori* hypothesized longitudinal contrasts (see dotted lines in Figure 4; Hupfeld et al., 2019; Salazar et al., 2020; McGregor et al., 2021). For these analyses, we also used SwE and TFCE toolboxes.

**Figure 3:**
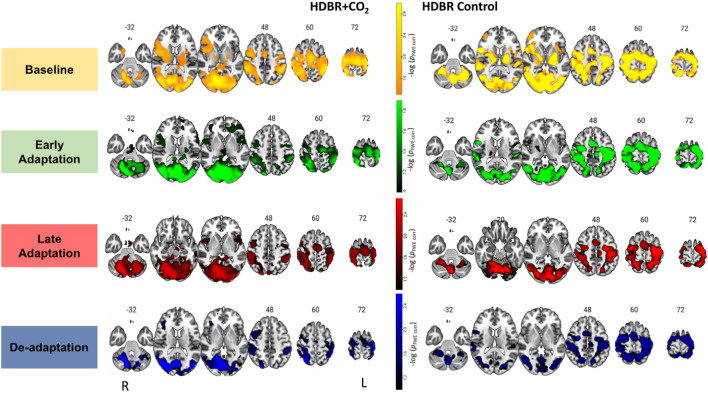
Brain activation during each phase of visuomotor adaptation. Here we depict the main effect for each group HDBR+CO_2_ (left) and HDBR Control (right) during each task phase: baseline (yellow-orange), early (green), late (red), and de-adaptation (blue). Whole brain results are overlaid onto an MNI standard template, thresholded at FWE < 0.10 and *k* = 10 voxels. The color scale depicts the -log(*p*_FWE-corr_) values in which brighter colors (higher values) represent smaller *p*-values.

**Figure 4:**
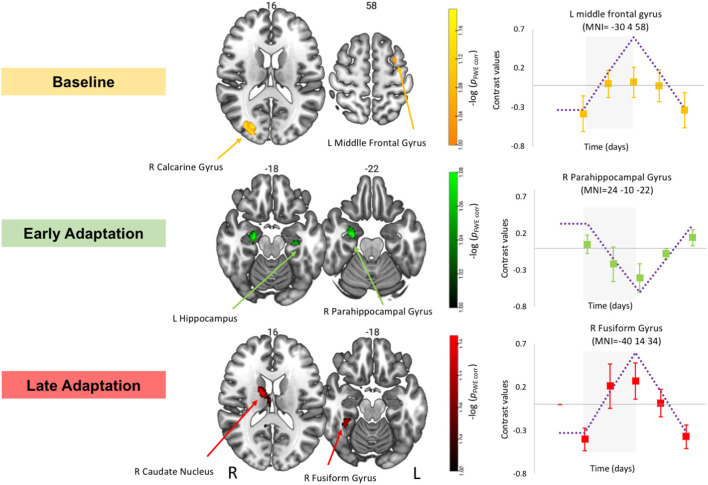
Time course of the neural visuomotor adaptation response to HDBR+CO_2_. Left: Whole brain results showing increases in activation during the baseline (yellow) and late phases of adaptation (red) and decreases in activation during early adaptation (green) followed by recovery. Whole brain results are overlaid onto an MNI standard template, thresholded at FWE < 0.10 and *k* = 10 voxels. The color scale depicts the -log(*p*_FWE-corr_) values in which brighter colors (higher values) represent smaller *p* values. Right: Example contrast values plotted for peak coordinate within the cluster with the smallest *p*-value in each case. Squares represent group mean contrast values; error bars represent standard error. Purple dotted lines show the hypothesized longitudinal contrasts for “cumulative decrease” and “cumulative increase” during-HDBR+CO_2_ followed by recovery. Gray box represents the intervention time in days.

#### Brain—Behavioral Correlations

To test for correlations between pre- to post-HDBR+CO_2_ brain changes and pre- to post- changes in direction error for each phase of the task we ran parametric one-sample t-tests in SPM12. We then re-estimated all models using the TFCE toolbox (Gaser, 2019) using all default settings, including 5,000 permutations.

#### HDBR+CO_2_ vs. HDBR Control Group Comparisons

As the HDBR+CO_2_ and HDBR control groups followed different testing timelines, we calculated the *slope* of change in brain activation with the HDBR intervention for each subject. Additionally, for each subject, we computed intercept images (i.e., baseline brain activation during each phase of visuomotor adaptation). In order to compare between-group slope changes accounting for baseline differences, we normalized the slope images using the formula: slope image/intercept image. We then performed two-sample t-tests for each phase of adaptation using the normalized slope images. Identical to above, we defined these models in SPM12 and re-estimated them using the TCFE toolbox (Gaser, 2019) with 5,000 permutations. These slope and intercept calculations are identical to those described in our past work (Yuan et al., 2018; Hupfeld et al., 2019; Salazar et al., 2020).

#### SANS vs. NoSANS Group Comparisons

To examine group differences between those subjects who developed signs of SANS (SANS; *n* = 5; two males, three females) and those who did not (NoSANS; *n* = 6; four males, two females) we performed one exploratory analysis for each phase of the task. We tested for differences between the normalized slope images for each group. We conducted two-sample parametric t-tests using the TFCE toolbox (Gaser, 2019).

We also performed a targeted follow-up analysis based on the behavioral results (Banker et al., 2021), in which we found a SANS vs. NoSANS difference in de-adaptation, i.e., SANS individuals showed larger direction error than the NoSANS participants. Also, in the post-HDBR+CO_2_ session, subjects with signs of SANS were slower to move the joystick (i.e., greater movement time and reaction time) during the de-adaptation blocks than those who did not develop signs of SANS. We calculated the average brain activation of the two post-HDBR+CO_2_ de-adaptation images. We then compared SANS vs. NoSANS differences using a two-sample t-test estimated with the TFCE toolbox (Gaser, 2019).

### Statistical Analyses of Behavioral Performance Data

We conducted the behavioral performance statistical analysis in R version 3.6.0 (IBM, Armonk, NY). We set alpha levels at 0.05 for the below models.

In order to test whether PaCO_2_ in the blood increased from pre- to post-HDBR+CO_2_, we performed a one-sample t-test.

Our group has previously reported detailed statistical analyses of the adaptation behavioral data for the HDBR+CO_2_ cohort (Banker et al., 2021). This previous article did not include behavior comparisons to the HDBR control group, as data collection for this cohort was not yet completed at the time of publication of our past work. Here, we compared between groups from two different bed rest campaigns (HDBR+CO_2_ and HDBR control), which were evaluated at different time points across their respective bed rest interventions. Thus, we entered time as a continuous variable to compare group effects. We included data from BR-7, BR7, and BR29 time points for the HDBR+CO_2_ group and BR-7, BR29, and BR58 time points for the HDBR control group. Linear mixed model analysis was used to examine group × block × time differences between HDBR+CO_2_ and HDBR subjects. The subject variable was entered as a random intercept. Age and sex were entered as covariates. There were no outliers or missing data among the three time points tested here.

## Results

There was no age difference between the HDBR+CO_2_ and HDBR control groups (*p* = 0.736). Similarly, there was no age difference between those participants who developed signs of SANS and those who did not (*p* = 0.925). No statistically significant difference in PaCO_2_ was observed from pre- (41.5 mmHg) to post- (42.8 mmHg) HDBR+CO_2_ (*p* = 0.120).

### Main Effect of Visuomotor Adaptation

Figure 3 depicts the average activation from each group and phase of adaptation compared to the rest. For the HDBR+CO_2_ group, main effect was averaged across all subjects (*n* = 11) and time points BR-7, BR7 and BR29. For the HDBR Control group main effect was averaged across all subjects (*n* = 8) and time points BR-7, BR29, and BR58. Brain activation patterns were visually similar between groups (Figure 3). As anticipated for these visuomotor adaptation phases (Ruitenberg et al., 2018), we observed task-related activation in frontal, occipital, sensorimotor, subcortical, and cerebellar regions during baseline, early, late adaptation, and de-adaptation.

### Time Course of Neural Visuomotor Adaptation Response to HDBR+CO_2_

#### Baseline

For the baseline trials (moving the cursor to targets with normal visual feedback), we found that brain activity increased with HDBR+CO_2_ in the right calcarine gyrus and left middle frontal gyrus (dorsal premotor cortex); these increases recovered towards the pre-intervention levels after subjects exited the HDBR+CO_2_ intervention (Figure 4; Table 1). We did not find decreases in brain activity in response to HDBR+CO_2_ for the baseline phase.

**Table 1:** Brain regions showing longitudinal increases and decreases in activation during visuomotor adaptation.

	TFCE-level			MNI coordinates (mm)		
	Extent (k_E_)	*p*_FWE-corr_	Contrast values	x	y	z
**Increases in activation**						
Baseline						
*Frontal*						
L Middle Frontal Gyrus	18	0.089	0.474	−30	4	58
*Occipital*						
R Calcarine Gyrus	222	0.064	0.908	22	−80	14
**Decreases in activation**						
Early Adaptation						
*Temporal*						
R Parahippocampal Gyrus	138	0.082	−0.502	24	−10	−22
L Hippocampus	182	0.083	−0.531	−26	−20	−14
*Subcortical*						
R Putamen	83	0.089	−0.490	30	−8	18
**Increases in activation**						
Late Adaptation						
*Temporal*						
R Fusiform Gyrus	99	0.075	0.565	−40	14	34
*Subcortical*						
R Caudate Nucleus	227	0.069	0.474	14	−4	18

*Note. Significance level set at FWE < 0.10 and cluster size *k* = 10 for all analyses. Brain regions labeled using the Anatomy Toolbox atlas *via* the SPM toolbox BSPMview. L = Left; R = Right. Degree of freedom: 8*.

#### Early Adaptation

We did not observe any increases in brain activity for the early adaptation blocks when subjects started HDBR+CO_2_. However, we observed decreasing activation in the right parahippocampal gyrus, right putamen, and left hippocampus for the early adaptation trials when subjects went into HDBR+CO_2_. These reductions in activation returned towards the pre- intervention levels at the two post-HDBR+CO_2_ time points (Figure 4; Table 1).

#### Late Adaptation

Across HDBR+CO_2_, we found increasing activation in the right fusiform gyrus and the right caudate nucleus for the late adaptation trial blocks; this activation then reduced when participants exited HDBR+CO_2_ (Figure 4; Table 1). We did not observe any decreases in brain activation for late adaptation.

#### De-adaptation

We did not observe any changes in brain activity during the de-adaptation phase in response to HDBR+CO_2_.

### Visuomotor Adaptation Behavioral Performance Results

We recently reported behavioral adaptation results during HDBR+CO_2_ for this cohort (Banker et al., 2021). In short, we found that HDBR+CO_2_ had no effect on baseline motor control when participants moved the joystick with normal visual feedback. During the early phase of adaptation, direction errors increased during HDBR+CO_2_ and then recovered post- HDBR+CO_2_. Participants presented with slower late adaptation reaction time post-HDBR+CO_2_ in comparison to the last day of bed rest; this finding was largely driven by the SANS subgroup (i.e., by those individuals who showed optic disc edema). Participants who developed signs of SANS also showed larger, more persistent aftereffects and were slower than the NoSANS group for reaction time and movement time post-intervention. Overall, there was a lack of savings of adaptation across multiple sessions. In the present work, we also compared performance metrics for the HDBR+CO_2_ group to those of the HDBR control group (in our previous work, data collection was not yet complete for the HDBR control group). Here we found a group by block by time interaction for baseline (*ß* = 0.131; *p* = 0.025) but not for the other parts of the task (early adaptation: *ß* = 0.048; *p* = 0.169; late adaptation: *ß* = 0.043; *p* = 0.218; de-adaptation: *ß* = 0.045; *p* = 0.578), suggesting that the behavioral effects we previously reported are largely due to HDBR as opposed to elevated CO_2_.

### Brain—Behavior Correlations

We did not find any brain-behavior correlations for any phase of the sensorimotor adaptation task.

### HDBR+CO_2_ vs. HDBR Control Group Comparisons

Between-group slope comparisons for HDBR+CO_2_ and HDBR control revealed non-significant differences in brain activity during all phases of the adaptation task.

### SANS vs. NoSANS

Between-subgroup slope comparisons for the SANS and NoSANS subgroups within the HDBR+CO_2_ cohort revealed no statistically significant differences in brain activity during any phase of the adaptation task. In addition, despite differences in behavioral performance between the SANS and NoSANS subjects during de-adaptation (Banker et al., 2021), we did not find any difference in brain activity when comparing the post-HDBR average between these two groups.

## Discussion

This pilot study investigated the effects of 30 days of strict HDBR combined with elevated CO_2_ levels on brain activation during a visuomotor adaptation task for 11 participants. In this cohort, we recently reported negative effects of HDBR+CO_2_ on adaptation savings (Banker et al., 2021); savings refers to the faster adaptation that occurs when a person repeats the same adaptation task. Previous work has shown that the magnitude of savings depends on the explicit, strategic components of adaptation (Taylor et al., 2014). The lack of savings showed in Banker et al. (2021) suggests that HDBR+CO_2_ induces a greater reliance on more procedural, implicit forms of adaptation. Here, we also observed decreases in brain activation in the right parahippocampal gyrus, right putamen, and left hippocampus during early adaptation after participants started HDBR+CO_2_. In contrast, for the late phase of adaptation, we found increases in temporal and subcortical brain regions in response to HDBR+CO_2_, followed by recovery by 2 weeks post-intervention. This is a small sample size pilot study and all the results reported here were corrected at FWE < 0.10.

### Main Effect of Visuomotor Adaptation

The main effect results of visuomotor adaptation were visually similar between the HDBR+CO_2_ and the control group. As anticipated (Ruitenberg et al., 2018), we observed widespread activation of frontal, temporal, occipital, subcortical, and cerebellar regions during all visuomotor adaptation phases. These results demonstrate that our visuomotor adaptation task produced the expected neural responses.

### Time Course of Visuomotor Adaptation Response to HDBR+CO_2_

During early adaptation, we identified decreasing activation in the right parahippocampal gyrus, right putamen, and left hippocampus after participants started HDBR+CO_2_. These changes normalized once participants exited the intervention. These brain regions are involved in a variety of cognitive processes, including long-term memory, visuospatial processing, and sensorimotor adaptation (Seidler et al., 2006; Toepper et al., 2010; Aminoff et al., 2013; Ni et al., 2017). We recently reported brain changes during a spatial working memory task in this same HDBR+CO_2_ cohort (Salazar et al., 2020). There, we found reduced brain activity in the right middle frontal gyrus and the cerebellar dentate nucleus across the HDBR+CO_2_ intervention followed by recovery, during the performance of the spatial working memory task (Salazar et al., 2020). Considering that early adaptation relies on spatial working memory processes (Anguera et al., 2010; Christou et al., 2016), we expected that both spatial working memory and visuomotor adaptation brain regions would overlap. However, our brain activity results show no overlap in brain regions affected by HDBR+CO_2_ regarding both tasks.

We found changes in hippocampus activity under two different tasks in the same cohort of HDBR participants. During visuomotor adaptation task participants showed decreases in activation in the parahippocampal and hippocampal. Our past work on spatial working memory brain changes found that participants who underwent 30 days of HDBR+CO_2_ presented greater decreases in activation in the right hippocampus than subjects who spent 70-days HDBR with normal CO_2_ atmospheric levels (Salazar et al., 2020). Previous work suggests that the hippocampus may be more sensitive to changes in CO_2_ compared to other brain regions (Scully et al., 2019). Moreover, a recent spaceflight analog study investigated the effects of social isolation and environmental deprivation and reported decreasing hippocampal volume in polar expeditioners who spent 14 months at the German Neumayer III station in Antarctica (Stahn et al., 2019). Thus, we believe that the decreases in activation observed in the present study during the early adaptation phase could have occurred at least in part as a result of participants spending 30 days in relative isolation and confinement. In addition, the various structural brain changes reported to occur with HDBR (e.g., Roberts et al., 2015) may also have contributed to the changes in brain function observed here.

It is widely accepted that early visuomotor adaptation is more cognitively demanding, whereas the late learning stage of a visuomotor adaptation task is more automatic or implicit (Anguera et al., 2010; Taylor et al., 2014). Here, during late adaptation participants showed increasing activation in the right fusiform gyrus and the right caudate nucleus during the late phase of adaptation in response to HDBR+CO_2_. In addition to the involvement of both of these structures in memory and learning (Grahn et al., 2008; Weiner and Zilles, 2016), the fusiform gyrus also plays a role in high-level visual processing and multisensory integration (Weiner and Zilles, 2016), while the caudate nucleus plays a role in planning the execution of movement, reward, and motivation (Grahn et al., 2008). Our results suggest that the HDBR+CO_2_ environment may cause a compensatory upregulation of brain activity to adapt to this phase of the task. Together, these early and late adaptation longitudinal brain changes suggest that HDBR+CO_2_ might lead to both neural adaptations and dysfunction.

### Visuomotor Adaptation Behavioral Results

In Banker et al. (2021), we reported that HDBR+CO_2_ changes the way in which individuals engage in sensorimotor processing. Specifically, after 30 days the HDBR+CO_2_ participants showed greater reliance on procedural, implicit processes during sensorimotor adaptation from pre- to post-intervention (Banker et al., 2021), evidenced by the lack of savings across sessions. In addition, during the early phase of adaptation, these participants showed greater direction error from pre- to post- intervention. They also presented with slower reaction time during the late phase of adaptation; this effect lasted from the last day of HDBR+CO_2_ throughout the two-week recovery period. It is possible that the sensorimotor reweighting effects of HDBR and the strict bed rest protocol contributed to this lack of savings.

Here we expanded on these past analyses by testing the isolated effect of CO_2_ by comparing HDBR+CO_2_ participants with a recently collected group of control subjects who underwent 60 days of HDBR without elevated CO_2_. We found a difference between the HDBR+CO_2_ and HDBR control groups during the baseline phase of the task. We were less focused on changes in basic motor control processes (as assayed by the baseline blocks) and more interested in the adaptation process. However, we did not find interactions in any of the other phases of the visuomotor task, suggesting that the effects on visuomotor adaptation performance are due more to HDBR than to elevated CO_2_, at least at the CO_2_ levels used here. Likewise, a recent study showed similar cognitive effects among the same cohort of participants examined here; i.e., HDBR+CO_2_ and HDBR control groups were not statistically different across a range of cognitive tasks that differed from the visuomotor adaptation task we reported on here (Basner et al., 2021). These complementary results suggest that—similar to what we report—elevated CO_2_ neither improved nor deteriorated the HDBR effects (Basner et al., 2021).

Regardless, it seems that the body unloading and/or the headward fluid shifts that occur with HDBR have an impact on sensorimotor adaptation behavior and brain activity. Both spaceflight and HDBR are known to result in sensory reweighting (Mulavara et al., 2018; Pechenkova et al., 2019). Here, it could be that sensory reweighting processes interfered with visuomotor adaptation, particularly in terms of savings.

### Brain—Behavior Correlations

To better understand the effects of an intervention such as HDBR on brain function, it is helpful to examine relationships between brain and behavior changes; this allows interpretation of whether identified brain changes are adaptive or dysfunctional (see Hupfeld et al., 2021). Here, we did not observe any brain-behavior change correlations, complicating interpretations. In this same cohort, we found evidence for both neural compensation and dysfunction as a result of HDBR+CO_2_ when participants performed a spatial working memory task (Salazar et al., 2020) or received vestibular stimulation (Hupfeld et al., 2019). However, the lack of associations found in the present study does not allow us to fully interpret whether identified longitudinal neural changes that occur during visuomotor adaptation across HDBR+CO_2_ are compensatory or dysfunctional. Taken together these results suggest that the compensatory and dysfunctional effects of a spaceflight analog with elevated atmospheric levels of CO_2_ could be task-specific rather than global effects of HDBR or CO_2_.

### HDBR+CO_2_ vs. HDBR Control

CO_2_ has a vasodilation effect which can result in increased intensity of the blood oxygen level-dependent signal measured by fMRI (Corfield et al., 2001) due to the increased brain blood flow (Atkinson et al., 1990; Zhou et al., 2008). In the present study, there were no increases in brain activity during visuomotor adaptation compared to HDBR alone. As we did not observe group differences in visuomotor adaptation performance between the HDBR+CO_2_ and HDBR control groups, we did not expect to find between-group differences in brain activation. We previously compared HDBR+CO_2_ and HDBR alone during a spatial working memory task (Salazar et al., 2020) and vestibular stimulation (Hupfeld et al., 2019). In our spatial working memory study, we found that the HDBR+CO_2_ participants had greater decreases in activation in the right hippocampus and left inferior temporal gyrus than in HDBR alone. Conversely, during vestibular stimulation, the HDBR+CO_2_ group showed greater increases in activation of several regions in comparison to HDBR alone (Hupfeld et al., 2019). These results suggest interactive or additive effects of HDBR+CO_2_ on vestibular processing (Hupfeld et al., 2019), spatial working memory (Salazar et al., 2020), but not visuomotor adaptation. Taken together, these findings support the idea that elevated CO_2_ effects may be task-specific, at least at the level and duration of elevated CO_2_ employed in this bed rest campaign.

### SANS vs. NoSANS

We had the unique opportunity to compare subgroups of those who did and did not develop signs of SANS. Behavioral results indicated that HDBR+CO_2_ influences the way SANS individuals engaged cognitive processes during adaptation; SANS subjects showed a greater reliance on procedural, implicit processes (Banker et al., 2021). Specifically, SANS individuals showed larger aftereffects during the de-adaptation phase (Banker et al., 2021). We also observed other behavioral differences between the SANS and NoSANS individuals (Lee et al., 2019a); those who developed signs of SANS increased their reliance on visual cues during HDBR+CO_2_, while NoSANS individuals remained less visually dependent (Lee et al., 2019a).

Contrary to our hypothesis, the present comparison between SANS and NoSANS revealed no statistically significant subgroup differences in brain activity during visuomotor adaptation. Previously we showed stronger correlations between pre- to post- HDBR+CO_2_ brain changes in vestibular processing with eyes open balance performance for subjects who developed signs of SANS compared to those who did not (Hupfeld et al., 2019), which further suggests that SANS may result in functional brain changes that impact behavioral performance. However, as the present work did not find any functional brain differences between SANS vs. NoSANS participants during sensorimotor adaptation, additional exploration of astronauts and spaceflight analog participants who do and do not develop SANS will help us to better understand resulting differences in brain function and possible implications for behavior.

### Limitations

The present study has several limitations. Due to the unique and difficult nature of HDBR interventions, we tested only a small pilot sample. The sample size and study durations for both the HDBR+CO_2_ and HDBR campaigns reported here were dictated by NASA and the European Space Agency (ESA). Given the small sample sizes, these results should be generalized with caution. We used a threshold of FWE < 0.10 for the neuroimaging statistical analyses to better detect within- and between-subject differences. Of note, we did not find any brain results at FWE < 0.05; as we used TFCE statistics, it was not possible to estimate the effect sizes of our results. In addition, our control group followed a different testing timeline from the HDBR+CO_2_ group; these groups were part of separate bed rest campaigns. However, these data were collected on the same scanner using identical fMRI sequences; we controlled our statistical analyses by using age and sex as nuisance covariates and (identical to our past work) by using slope comparisons to account for differences in testing timelines (Yuan et al., 2016, 2018; Hupfeld et al., 2019; Salazar et al., 2020). Also, the small group sizes of *n* = 11 and *n* = 8 probably reflected in the lack of statistical differences between groups, suggesting that larger studies comparing HDBR+CO_2_ and HDBR alone are needed to more precisely understand the effects of CO_2_. Finally, HDBR+CO_2_ models only some of the effects of spaceflight such as elevated CO_2_ levels, body unloading, and fluid shifts towards the head. Moreover, astronauts are faced with additional factors such as more prolonged isolation, disrupted sleep and circadian cycles, radiation, etc (Clement et al., 2020). Therefore, it is difficult to generalize HDBR findings broadly to spaceflight. We are currently studying this visuomotor adaptation task in an ongoing prospective study involving astronauts completing six-month ISS missions (Koppelmans et al., 2013). Future work will compare behavioral and brain changes that occur during spaceflight with these HDBR+CO_2_ data to aid interpretations and better understand the accuracy of HDBR+CO_2_ as an analog for spaceflight effects on visuomotor adaptation.

## Conclusions

We investigated the longitudinal neural effects of HDBR+CO_2_ on visuomotor adaptation. We observed decreases in activation in several brain regions involved in long-term memory and visuospatial processing; these changes were resolved by 2 weeks after the conclusion of HDBR+CO_2_. This suggests that 30 days of HDBR combined with elevated CO_2_ levels may reduce one’s ability to recruit these brain regions. In addition, during late adaptation we observed increases in activation in the right fusiform gyrus and the right caudate nucleus, indicating a possible compensatory brain response to the HDBR+CO_2_ environment. A lack of brain-behavior correlations complicates this interpretation, however, and thus further work in larger samples is needed. Together, these results contribute to a better understanding of how the visuomotor system adapts to a spaceflight analog environment.

## Data Availability Statement

The raw data supporting the conclusions of this article will be made available by the authors, without undue reservation.

## Ethics Statement

The studies involving human participants were reviewed and approved by the local ethical commission of the regional medical association in Germany (Ärztekammer Nordrhein) and by the University of Florida and NASA Institutional Review Boards. The patients/participants provided their written informed consent to participate in this study.

## Author Contributions

AS analyzed the visuomotor adaptation fMRI data, created the figures and tables, and wrote the first draft of the manuscript. KH assisted with fMRI preprocessing and preparation of the initial manuscript draft. JL and EM collected, analyzed, and managed data. LB and GT analyzed the behavioral data. NB collected and analyzed data. IK participated in project design and software development. YD collected and analyzed data. JB, AM, and RS designed the project, secured funding, and led the interpretation and discussion of the results. All authors participated in revision of the manuscript. All authors contributed to the article and approved the submitted version.

## Conflict of Interest

NB, IK, YD, and AM were employed by the company KBR. The remaining authors declare that the research was conducted in the absence of any commercial or financial relationships that could be construed as a potential conflict of interest.

## Footnotes

^1^www.nitrc.org/projects/artifact_detect/
