# Supplementary material for: Visuomotor Adaptation Brain Changes During a Spaceflight Analog With Elevated Carbon Dioxide (CO2): A Pilot Study
Source: Front Neural Circuits. 2021 Jun 7;15:659557. doi: 10.3389/fncir.2021.659557 (PMC8215599; doi:10.3389/fncir.2021.659557)
Supplement: Supplementary file 1 [file Table_1.docx]

**Supplementary table** | Exploratory (uncorrected) results to provide data for future power analyses or planned region of interest analyses.

*Note:* None of the voxelwise comparisons (brain-behavioral correlations, HDBR+CO_2_ vs HDBR Control, and SANS vs NoSANS) were significant at FWE 0.1 or 0.05.

**Brain – Behavior Correlations**

|  | Extent (k_E_) | MNI coordinates (mm)  (x, y, z) | p-uncorrected | |
| --- | --- | --- | --- | --- |
| **Baseline** |  |  |  |  |
| Positive |  |  |  |  |
| L Fusiform Gyrus | 263 | -24, -17, -30 | <0.001 |  |
| R Cerebellum (Crus 2) | 30 | 56, -62, -35 | <0.001 |  |
| R Thalamus | 351 | 9, -30, 6 | <0.001 |  |
| R Rolandic Operculum | 114 | 47, -12, 14 | <0.001 |  |
| R SupraMarginal Gyrus | 64 | 43, -33, 39 | <0.001 |  |
| R Superior Medial Gyrus | 41 | 10, 47, 41 | <0.001 |  |
| L Precentral Gyrus | 120 | -43, -5, 38 | <0.001 |  |
| L Inferior Temporal Gyrus | 54 | -48, -13, -33 | <0.001 |  |
| L Precuneus | 25 | -6, -78, 55 | <0.001 |  |
| Negative |  |  |  |  |
| L Middle Orbital Gyrus | 396 | -27, 49, -10 | <0.001 |  |
| R Linual Gyrus | 339 | 29, -93, -12 | <0.001 |  |
| L Cerebelum (Crus 1) | 134 | -39, -63, -29 | <0.001 |  |
| R Superior Occipital Gyrus | 282 | 24, -85, 33 | <0.001 |  |
| R Precuneus | 133 | 8, -61, 54 | <0.001 |  |
| L Linual Gyrus | 187 | -14, -104, -9 | <0.001 |  |
| R Superior Frontal Gyrus | 23 | 19, 5, 59 | <0.001 |  |
| R Middle Occipital Gyrus | 37 | 38, -88, 11 | <0.001 |  |
| R Posterior-Medial Frontal | 45 | 15, -2, 63 | 0.001 |  |
| R Middle Occipital Gyrus | 13 | 32, -71, 32 | <0.001 |  |
|  |  |  |  |  |
| **Early Adaptation** |  |  |  |  |
| Negative |  |  |  |  |
| R ParaHippocampal Gyrus | 193 | 37, -24, -18 | <0.001 |  |
| R Anterior Cingulate | 414 | 3, -2, 34 | <0.001 |  |
| L Inferior Frontal Gyrus | 891 | -49, 23, 17 | <0.001 |  |
| L Fusiform Gyrus | 125 | -32, -43, -14 | <0.001 |  |
| R Inferior Frontal Gyrus | 26 | 21, 13, -20 | <0.001 |  |
| L Calcarine Gyrus | 80 | -6, -96, -7 | <0.001 |  |
| R Olfactory cortex | 34 | 3, 21, 0 | <0.001 |  |
| R SupraMarginal Gyrus | 164 | 45, -46, 47 | <0.001 |  |
| L ParaHippocampal Gyrus | 22 | -19, -2, -18 | <0.001 |  |
| R Superior Parietal Lobule | 53 | 21, -57, 68 | <0.001 |  |
| R Linual Gyrus | 48 | 18, -91, -7 | <0.001 |  |
|  |  |  |  |  |
| **Late Adaptation** |  |  |  |  |
| Positive |  |  |  |  |
| L Caudate Nucleus | 91 | -14, 6, 25 | <0.001 |  |
| Negative |  |  |  |  |
| R Precentral Gyrus | 330 | 28, -28, 74 | <0.001 |  |
| L Inferior Frontal Gyrus | 139 | -56, 11, 11 | <0.001 |  |
| R Cerebellum (VIII) | 493 | 22, -75, -47 | <0.001 |  |
| R Putamen | 684 | 37, -6, 3 | <0.001 |  |
| R Postcentral Gyrus | 19 | 26, -41, 70 | <0.001 |  |
| L Medial Temporal Pole | 20 | -43, 17, -32 | <0.001 |  |
| R Middle Temporal Gyrus | 13 | 61, -13, -7 | 0.001 |  |
|  |  |  |  |  |
| **De-adaptation** |  |  | <0.001 |  |
| L Cerebellum (VIII) | 15 | -14, -74, -46 | <0.001 |  |

*Note:* This table presents the clusters that show correlations with changes in behavior (direction error) during visuomotor adaptation. Cluster size k=10 for all analyses. Brain regions labeled using the AnatomyToolbox atlas via the SPM toolbox BSPMview. L= Left; R= Right.

**HDBR+CO_2_ vs HDBR Control**

|  | Extent (k_E_) | | MNI coordinates (mm)  (x, y, z) | p-uncorrected | |
| --- | --- | --- | --- | --- | --- |
| **Baseline** |  | |  |  |  |
| HDBR+CO_2_ < HDBR Control | |  |  |  |  |
| R Inferior Temporal Gyrus | 22 | | 46, -14, -40 | <0.001 |  |
| R Rectal Gyrus | 12 | | 6, 58, -14 | <0.001 |  |
| R Cerebelum (Crus 2) | 11 | | 56, -60, -38 | <0.001 |  |
|  |  | |  |  |  |
| **Early Adaptation** |  | |  |  |  |
| HDBR+CO_2_ < HDBR Control |  | |  |  |  |
| L Temporal Pole | 12 | | -24, 16, -28 | <0.001 |  |
| L Cerebelum (Crus 1) | 12 | | -52, -58, -36 | <0.001 |  |
|  |  | |  |  |  |
| **Late Adaptation** |  | |  |  |  |
| HDBR+CO_2_ < HDBR Control |  | |  |  |  |
| L Superior Frontal Gyrus | 13 | | -26, 38, 54 | <0.001 |  |
| L Medial Temporal Pole | 13 | | -18, 12, -36 | <0.001 |  |
| **De-adaptation** |  | |  |  |  |
| HDBR+CO_2_ < HDBR Control |  | |  |  |  |
| R Superior Medial Gyrus | 11 | | 12, 30, 56 | <0.001 |  |
| L Calcarine Gyrus | 12 | | 2, -102, -2 | <0.001 |  |
| L Superior Medial Gyrus | 22 | | 2, 26, 56 | <0.001 |  |
| L Middle Frontal Gyrus | 12 | | -42, 6, 62 | <0.001 |  |
| R Cerebellum (Crus 2) | 16 | | 12, -96, -22 | <0.001 |  |
| L Cuneus | 18 | | 4, -98, 22 | <0.001 |  |
| R Cerebelum (Crus 1) | 29 | | 28, -92, -26 | <0.001 |  |

*Note:* This table presents the clusters that show difference between HDBR+CO_2_ vs HDBR Control group during visuomotor adaptation. Cluster size k=10 for all analyses. Brain regions labeled using the AnatomyToolbox atlas via the SPM toolbox BSPMview. L= Left; R= Right.

**SANS vs NoSANS**

|  | Extent (k_E_) | MNI coordinates (mm)  (x, y, z) | p-uncorrected | |
| --- | --- | --- | --- | --- |
| **Baseline** |  |  |  |  |
| SANS < NoSANS |  |  |  |  |
| L Cerebelum (IV-V) | 20 | -24, -42, -24 | <0.001 |  |
| R Cerebellum (VI) | 21 | 16, -74, -14 | <0.001 |  |
|  |  |  |  |  |
| **Early Adaptation** |  |  |  |  |
| SANS < NoSANS |  |  |  |  |
| L Middle Temporal Gyrus | 24 | -62, -20, -10 | <0.001 |  |
|  |  |  |  |  |
| **Late Adaptation** |  |  |  |  |
| SANS > NoSANS |  |  |  |  |
| L Superior Orbital Gyrus | 10 | -16, 56, -4 | <0.001 |  |
| SANS < NoSANS |  |  |  |  |
| L Middle Occipital Gyrus | 19 | -22, -104, 2 | <0.001 |  |
| L Superior Medial Gyrus | 13 | -6, 28, 50 | <0.001 |  |
| **De-adaptation** |  |  |  |  |
| SANS < NoSANS |  |  |  |  |
| L Inferior Temporal Gyrus | 15 | -36, -44, -10 | <0.001 |  |
| R Cerebelum (VI) | 23 | 18, -62, -16 | <0.001 |  |

*Note:* This table presents the clusters that show difference between SANS vs NoSANS subgroup during visuomotor adaptation. Cluster size k=10 for all analyses. Brain regions labeled using the AnatomyToolbox atlas via the SPM toolbox BSPMview. L= Left; R= Right.
